# Supplementary material for: Epigenome-wide DNA methylation profiling in Progressive Supranuclear Palsy reveals major changes at DLX1
Source: Nat Commun. 2018 Jul 26;9:2929. doi: 10.1038/s41467-018-05325-y (PMC6062504; doi:10.1038/s41467-018-05325-y)
Supplement: Supplementary file 1 — Supplementary Information [file 41467_2018_5325_MOESM1_ESM.pdf]

<u>Supplementary Information</u>

# **Epigenome-wide DNA methylation profiling in Progressive Supranuclear Palsy reveals major changes at *DLX1***

Weber et al.

Supplementary Figure 1

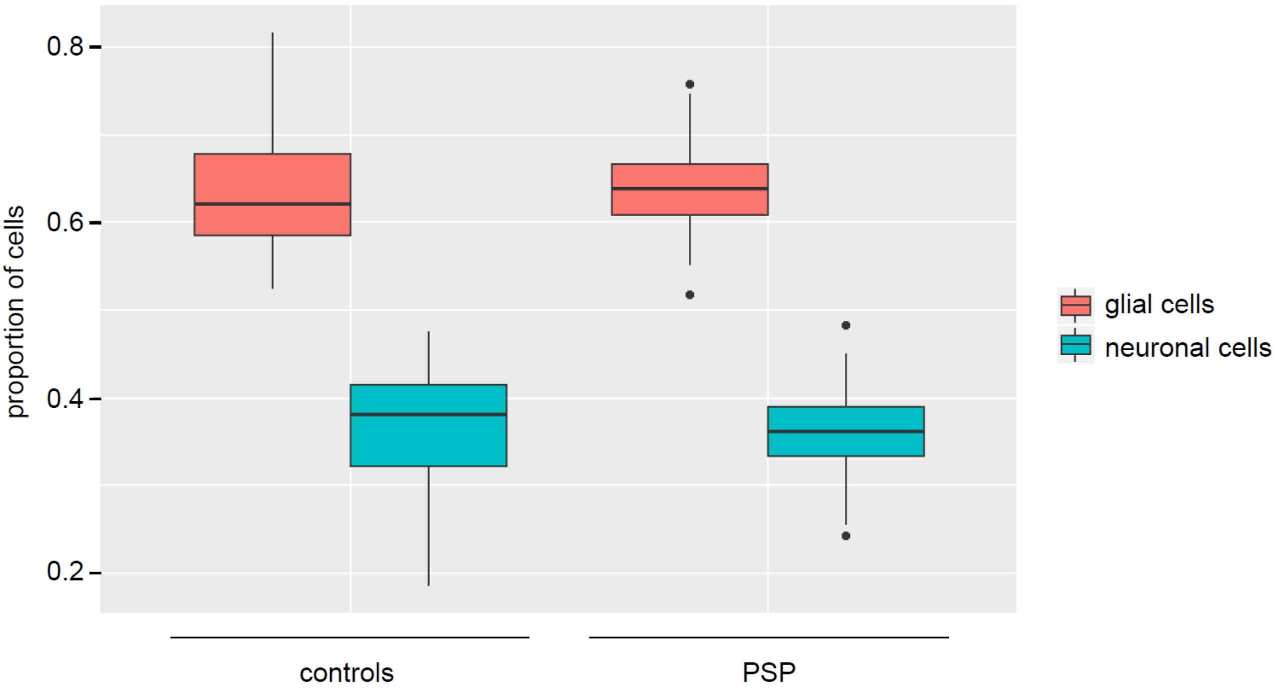


**Supplementary Figure 1 | Proportion of celltypes in samples of prefrontal cortex of controls and PSP patients.**

Proportion of neuronal and non-neuronal (glial) cells in PSP and control prefrontal cortex as estimated by the "estimate CellCounts" function of the mini Bioconductor package using dorsolateral prefrontal cortex as composite cell type. Although there is - as expected- a slight decrease in neuronal cell content in PSP brains (n=94) as compared to control brains (n=71), the difference did not reach statistical significance (Wilcoxon Test, P=0.31). The line in the middle of the box and whisker graph represents the median ($50^{th}$ percentile). The box extends from the $25^{th}$ to $75^{th}$ percentile. The whiskers extend down to the 5% percentile value and up to the 95% percentile value.

Supplementary Figure 2

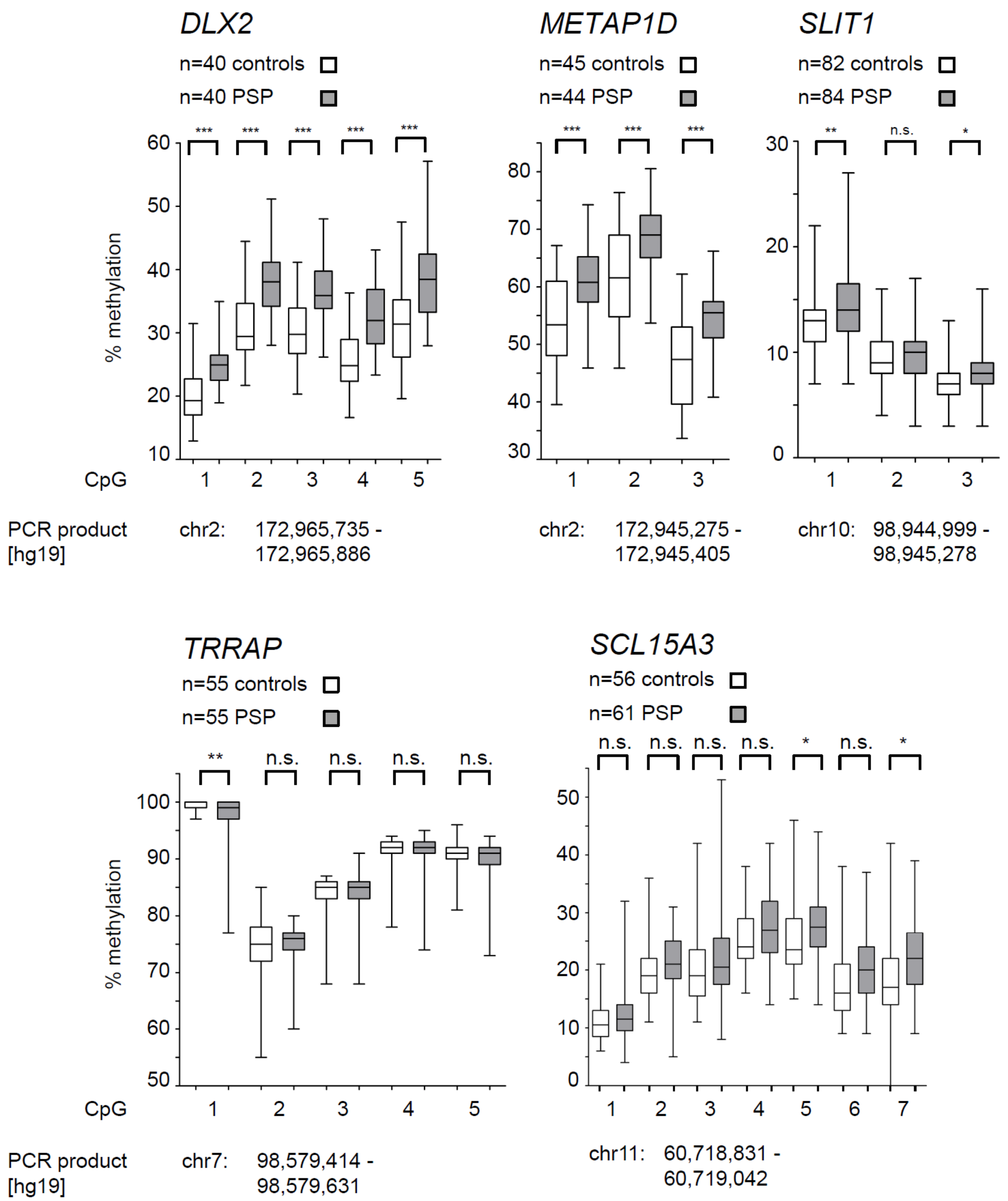


**Supplementary Figure 2 | Validation of differentially methylated genes.**
Differential methylation indicated by BeadChip analysis was confirmed by pyrosequencing for the following genes: 1) *DLX2* (5 CpG sites), *METAP1D* (3 CpG sites), *SLIT1* (2 CpG sites), *TRRAP* (1 CpG site), *SCL15A3* (2 CpG sites). The line in the middle of the box and whisker graph is the median (50th percentile). The box extends from the 25th to 75th percentile. The whiskers extend down to the lowest value and up to the highest. Statistics for each CpG site are given in a separate table under the graph. Welchs's corrected t-test was used for data which are normally distributed in nearly all cases of CpG-control and CpG-PSP samples according to Kolmogorov-Smirnov. For data which are not normally distributed the Mann-Whitney test was used. *P<0.05, **P<0.01, ***P<0.001, n.s. = not significant.

Supplementary Figure 3

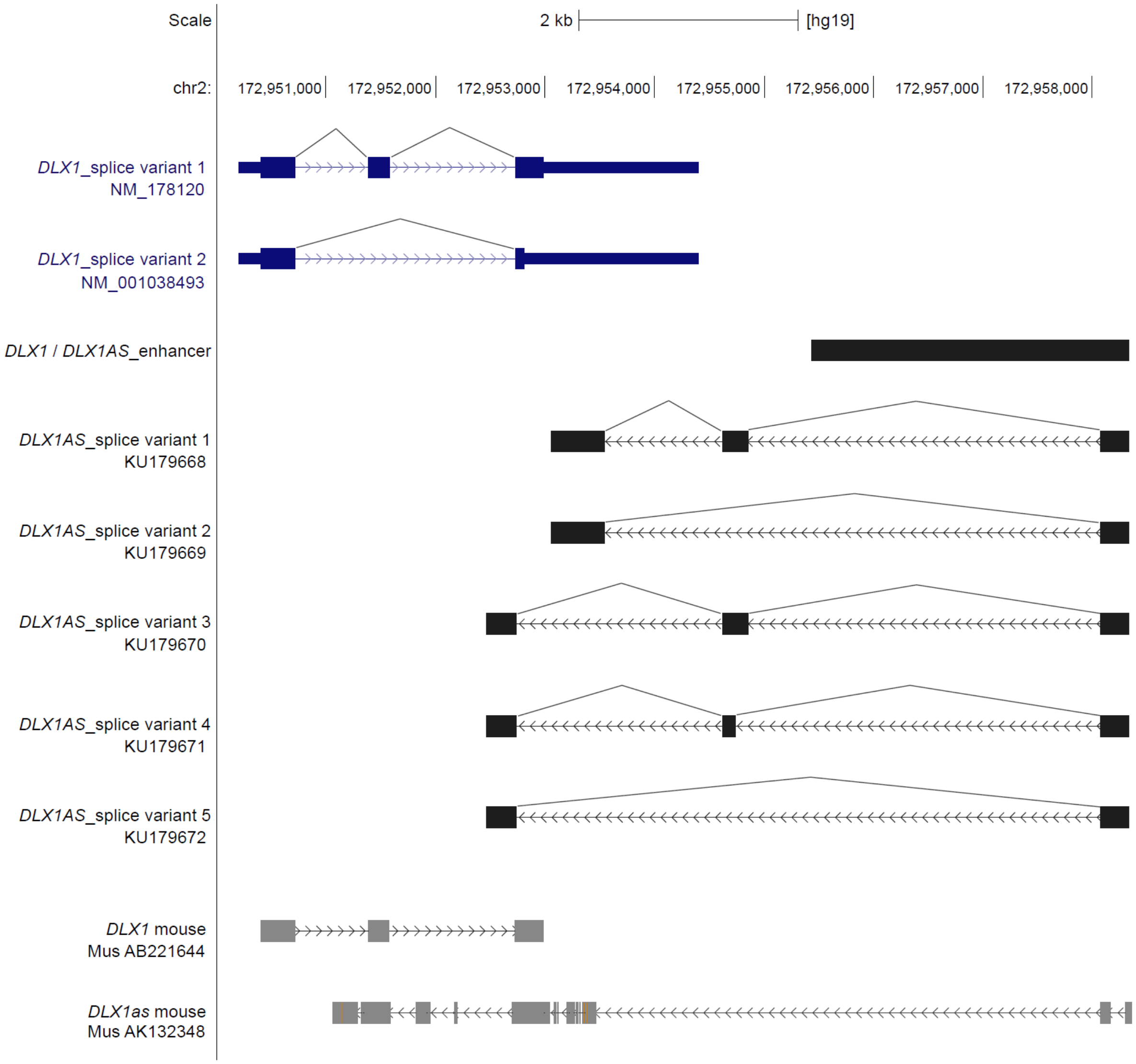


**Supplementary Figure 3 | Genomic structure of *DLX1* and *DLX1AS* and their splice variants.**
NCBI numbers have been assigned and are given on the left. The *DLX1* enhancer is shown, as described[19].

Supplementary Figure 4

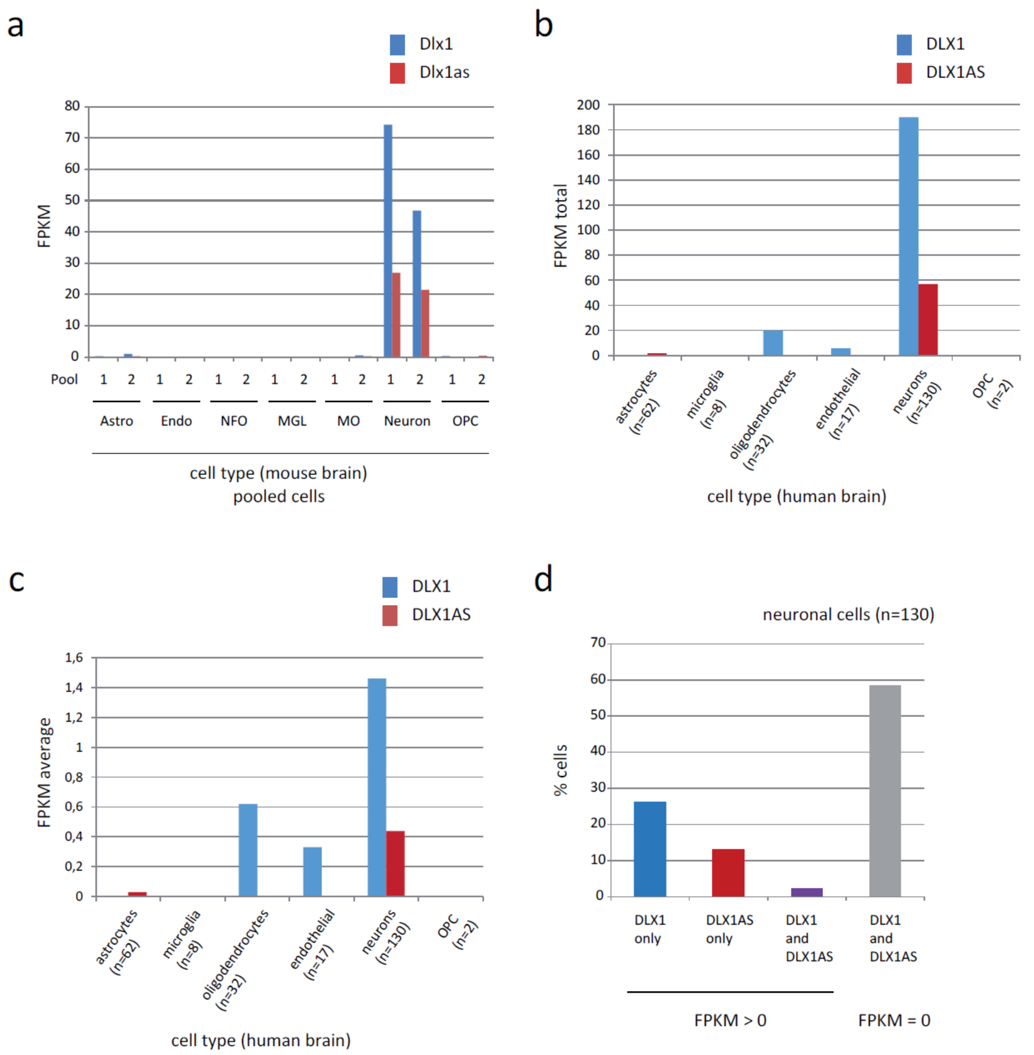


**Supplementary Figure 4 | Expression of DLX1 and DLX1AS Transcripts**
(**a**) Expression of *Dlx1* (blue) and *Dlx1as* (red), displayed as fragments per kilobase of transcript sequence per million mapped fragments (FPKM), in different cell types of mouse brain tissue [astrocytes (Astro), endothelial cells (Endo), newly formed oligodendrocytes (NFO), microglia (MGL), myelinating oligodendrocytes (MO), neuronal cells (Neuron), oligodendrocyte precursor cells (OPC); data from Zhang et al.[22]] Both *Dlx1* and *Dlx1as* transcripts are almost exclusively expressed in neurons in the mouse brain.
(**b**) Total number of reads aligned to transcript sequences over all cells for the corresponding type of either *DLX1* (blue) or *DLX1AS* (red) from healthy human cerebral cortex (data from Darmanis et al.[23]). Reads have been corrected for sequence depth of each cell and sequence length of each transcript and are displayed as FPKM. Both *DLX1* and *DLX1AS* transcripts are almost exclusively expressed in neurons in the human cerebral cortex.
(**c**) Average number of reads per cell (mean) of either *DLX1* (blue) or *DLX1AS* (red) from healthy human cerebral cortex (Data from Darmanis et al.[23]). Total number of reads of either *DLX1* or *DLX1AS* from cells of a specific type (e.g. neurons) have been divided by the total number of these cells. Reads have been corrected for sequence depth of each cell and sequence length of each transcript and are displayed as FPKM.
(**d**) N=130 individual neurons of healthy human cerebral cortex were tested for expression of *DLX1* or *DLX1AS*. This analysis showed that neurons either expressed *DLX1* blue) or *DLX1AS* red), or none of both (grey). Only 2.31% of neurons express both *DLX1* and *DLX1AS* (purple).

Supplementary Figure 5

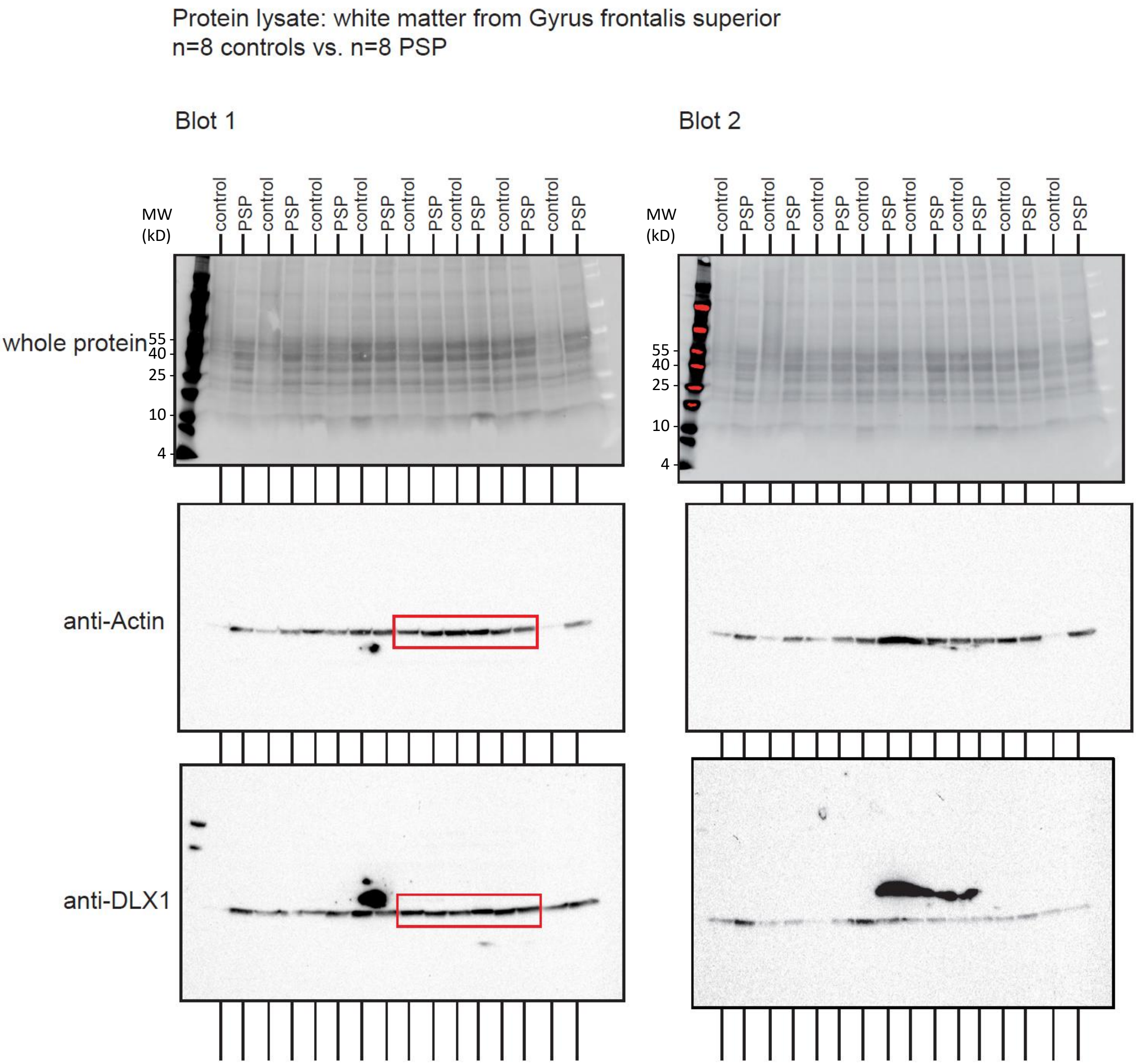


**Supplementary Figure 5 | DLX1 Western blots of white matter from gyrus frontalis superior.**

DLX1 protein in white matter of gyrus fronalis superior as detected on uncropped full length Western blots (N=8 PSP and N=8 controls) Blot 1 and 2 are technical replicates. 2,2,2-Trichloroethanol labelled total protein and the housekeeping protein β-actin were used as loading control. The boxed area (red) is shown as representative selection in Fig. 4c.

Molecular weight markers used: lane 1 #830537 and lane 18 #830552 (Hessisch Oldendorf, Germany)

Supplementary Figure 6

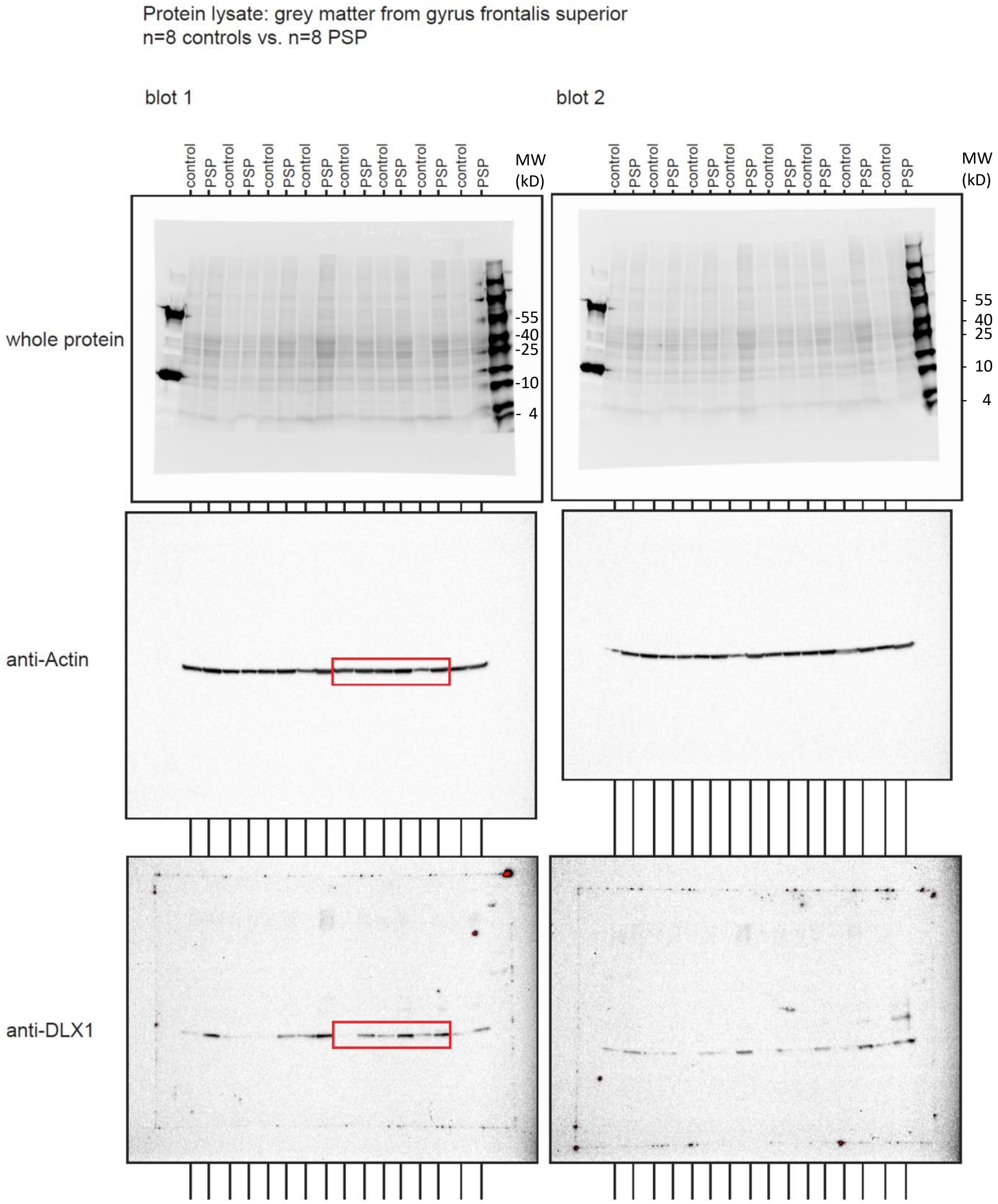


**Supplementary Figure 6 | DLX1 Western blots of grey matter from gyrus frontalis superior.**

DLX1 protein in grey matter of gyrus fronalis superior as detected on uncropped full length Western blots (N=8 PSP and N=8 controls). Blot 1 and 2 are technical replicates. 2,2,2-Trichloroethanol labelled proteins and the housekeeping protein β-actin were used as loading control. The boxed area (red) is shown as representative selection in Fig. 4d.

Molecular weight markers used: lane 1 #830552 and lane 18 #830537 (Hessisch Oldendorf, Germany)

Supplementary Figure 7

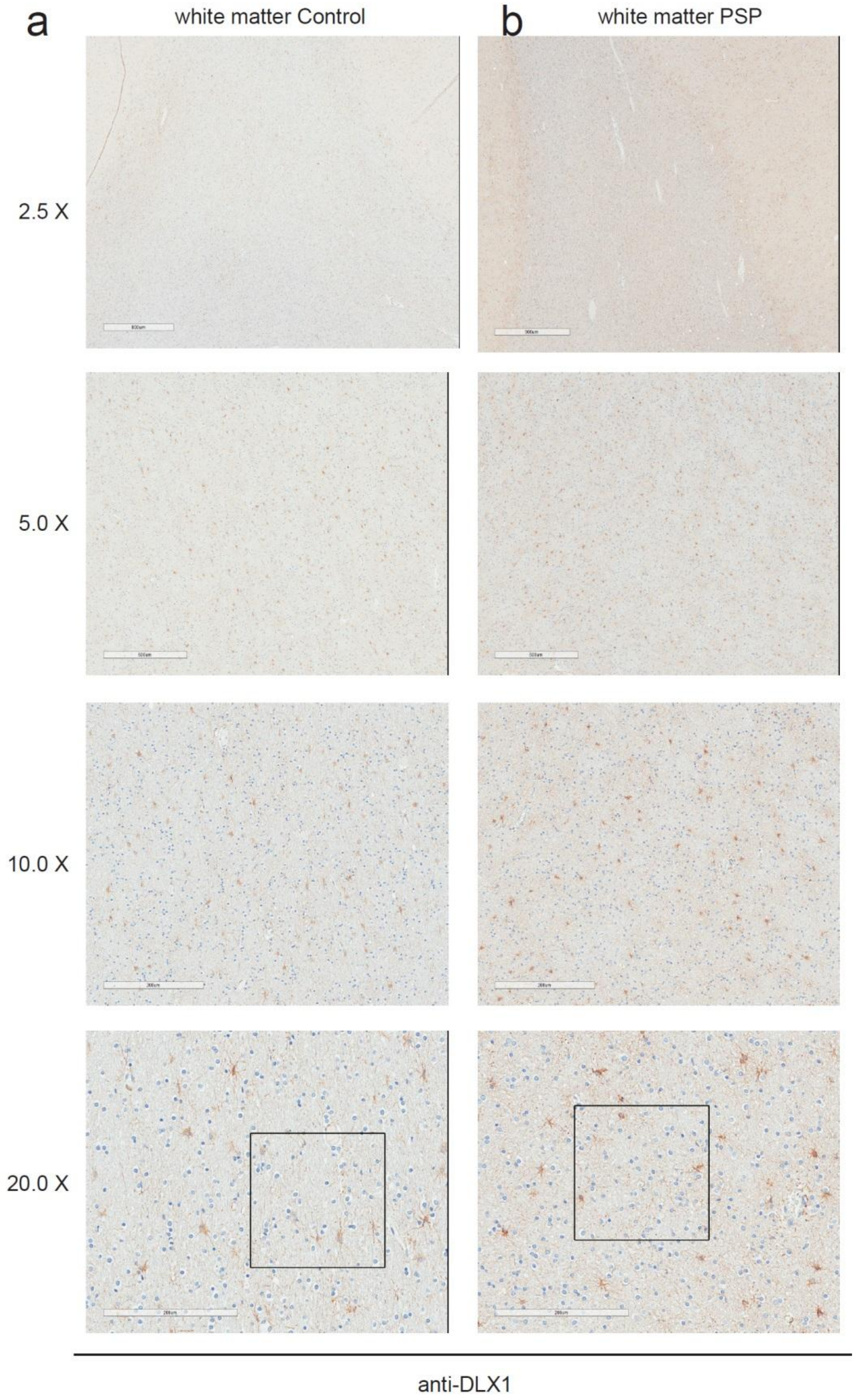


**Supplementary Figure 7 | DLX1 immunohistochemistry of white matter of gyrus frontalis superior.**

Uncropped DLX1-immunostaining of histological sections of white matter of the gyrus frontalis superior derived from a representative control and a PSP patient. Different magnifications are shown. The boxed area is depicted as representative selection in Fig. 4e.

Supplementary Figure 8

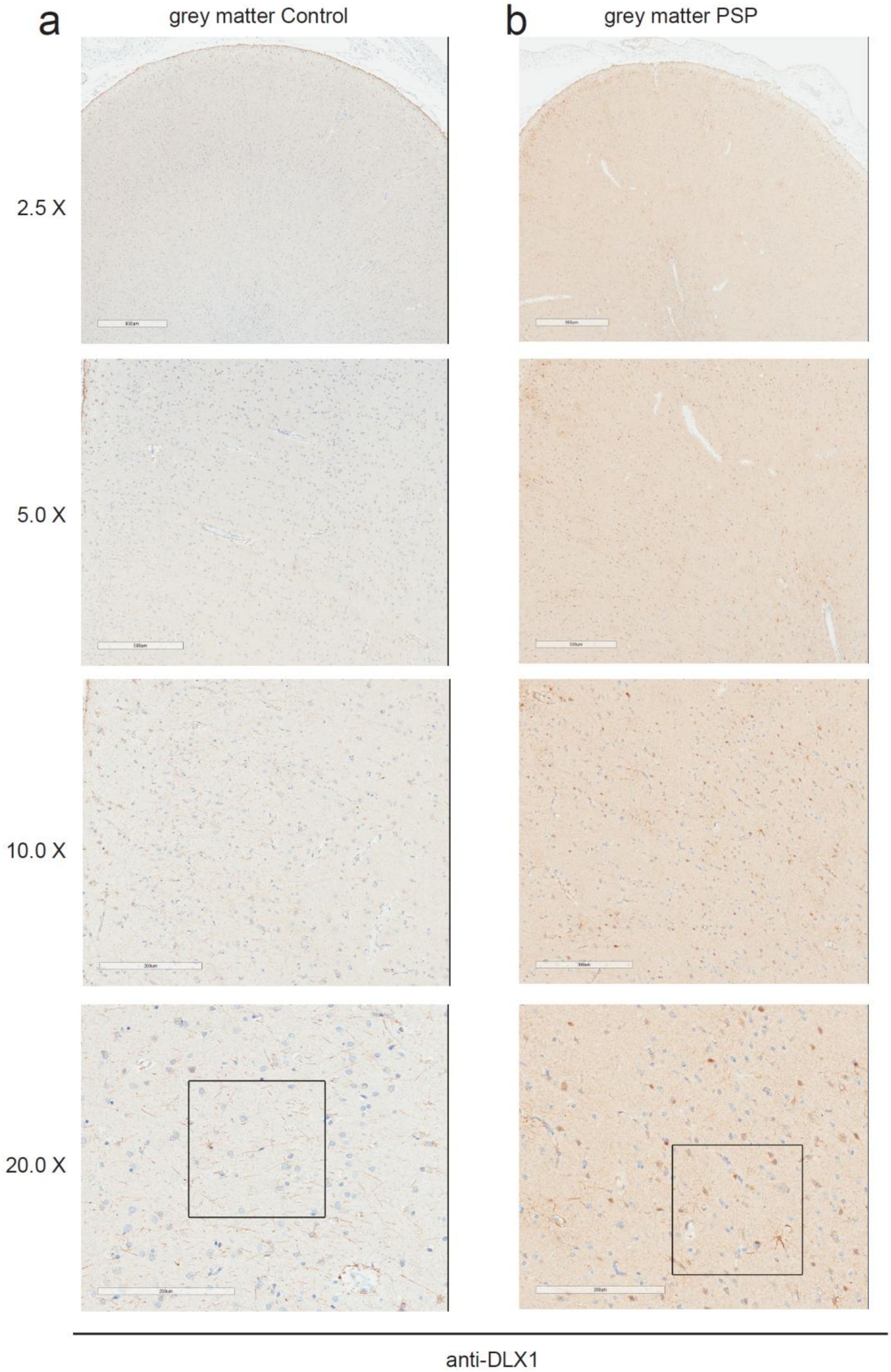


**Supplementary Figure 8 | DLX1 immunohistochemistry of grey matter of gyrus frontalis superior.**

Uncropped DLX1-immunostaining of histological sections of grey matter of the gyrus frontalis superior derived from a representative control and a PSP patient. Different magnifications are shown. The boxed area is depicted as representative selection in Fig. 4f.

Supplementary Figure 9

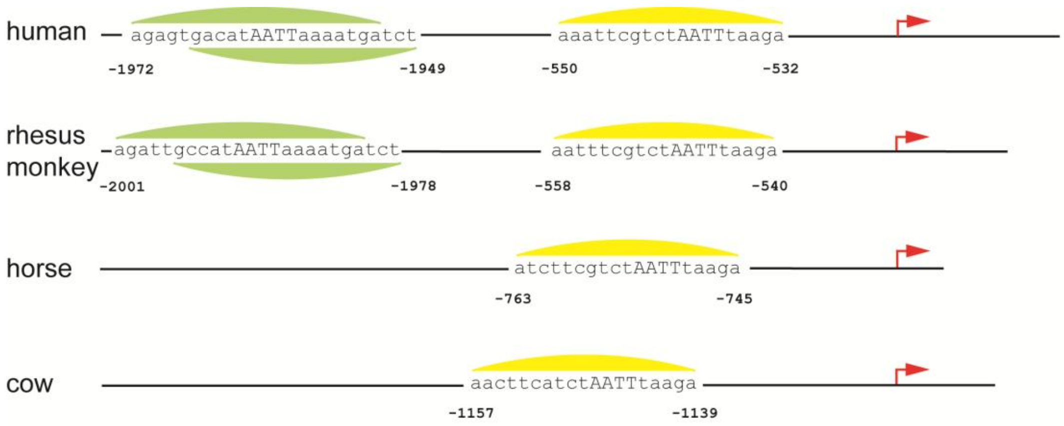


**Supplementary Figure 9: In-silico promoter analysis of *MAPT***

Schematic representation of *MAPT* promoter sequences from human (Homo sapiens), rhesus monkey (Macaca mulatta), horse (Equus caballus) and cow (Bos taurus). The red arrow indicates the transcription start side (TSS), and the positions are denoted relative to the TSS. Predicted DLX1 binding sites (V$DLX1.01) are represented by semicircles. Green DLX1 binding sites located on both strands were found conserved across human and rhesus monkey with help of the DiAlignTF program whereas the yellow DLX1 binding site is detected in the promoter sequences of human, rhesus monkey, horse and cow (optimized matrix threshold of V$DLX1.01: 0.91 (-0.02)).

# Supplementary Table 1

| Primer used for | Gene | | | Sequence |
|---|---|---|---|---|
| Pyrosequencing | | | | |
| | SLC15A3 | F | | AGTTGGTGTTGTTGAGGTATAG |
| | SLC15A3 | R | biotinylated | AACCCAACCCRAAACCAAAATACC |
| | SLC15A3 | SEQ | | GTGTTGTTGAGGTATAGT |
| | SLIT1 | F | | AGGTGTGAGGAYGGAAGTTGATAATT |
| | SLIT1 | R | biotinylated | ACTACAAACCATTCCCAAAAATATAC |
| | SLIT1 | SEQ | | GTATTGATTYGTTTGTGGGT |
| | TRRAP | F | | AGATGGTTTGGAAGGTGAATAT |
| | TRRAP | R | biotinylated | ACTACCACAAACCACAAACCTATACT |
| | TRRAP | SEQ | | GTTTTATAGAGAGTTTGGTGGAGA |
| | DLX2 | F | | AGAGGAGAAAAAAGAAGGTAGAG |
| | DLX2 | R | biotinylated | ACCAACAATCCTAACCTTTTAACAATTAC |
| | DLX2 | SEQ | | GGGGAGATAAGAGGAT |
| | METAP1D | F | biotinylated | GGGGGAGATTGAAGAGTAA |
| | METAP1D | R | | TCCTCCAATCCCCTCCAAA |
| | METAP1D | SEQ | | CCAAAACCAAAATTTATTT |
| | DLX1 | F | | AGAAGGATTTAGAGGGAAGAAGGAATAG |
| | DLX1 | R | biotinylated | AACCACTCTCTACTACCACTACT |
| | DLX1 | SEQ | | GGGAAGAAGGAATAGT |
| Gene expression Analysis | | | | |
| RT-qPCR (pimary tissue) | gene (primer name) | | | primer-sequence (5´-3´) |
| | EIF4 A2 | F | | ATACGAGGCGCAAGGTG |
| | EIF4 A2 | R | | ACTTGTTGCACATCAATCCC |
| | CYC1 | F | | AGTTTGACGATGGCACCC |
| | CYC1 | R | | GCCGCTTTATGGTGTAGAC |
| | DLX1 | F | | GTTTATGGAGTTTGGGC |
| | DLX1 | R | | AGTGTAAACAGTGCATGGAGTA |
| | DLX1AS | F | | GATAGGAGGATGGGTCTG |
| | DLX1AS | R | | TGGACACACACTCTTTGC |
| RT-qPCR (cell culture, SH-EP, NT2) | B2M | F | | TCTCGCTCCGTGGCCT |
| | B2M | R | | TCAGTAAGTCAACTTCAATGTCGGAT |
| | DLX1AS | F | | CAAAAGCTACCTTCGACCCT |
| | DLX1AS | R | | TGGACACACACTCTTTGC |
| | MAPT | F | | CCATGCCAGACCTGAAGAA |
| | MAPT | R | | CACACTTGGACTGGACGTT |
| | GAD1 | F | | CATCTTCGTCCGCAACCTC |
| | GAD1 | R | | GCACTCACAAGGCGACTCT |
| | BRN3B | F | | ATGCGGAGAGCCTGTCTTC |
| | BRN3B | R | | CTCTGGGAGACGATGTCCAC |
| | OLIG2 | F | | CCTGAGGCTTTTCGGAGC |
| | OLIG2 | R | | GATAGTCGTCGCAGCTTTCG |

**Supplementary Table 1 | Primers.**
The table provides sequence information on primers used for pyrosequencing and RT-qPCR
