# Supplementary material for: Epigenome-wide DNA methylation profiling in Progressive Supranuclear Palsy reveals major changes at DLX1
Source: Nat Commun. 2018 Jul 26;9:2929. doi: 10.1038/s41467-018-05325-y (PMC6062504; doi:10.1038/s41467-018-05325-y)
Supplement: Supplementary file 3 — Description of Additional Supplementary Files [file 41467_2018_5325_MOESM3_ESM.pdf]

Description of Additional Supplementary Files

File Name: Supplementary Data 1
Description: Information on clinical and neuropathological findings in PSP patients and controls.

File Name: Supplementary Data 2
Description: Differentially methylated CpG sites in PSP vs. controls.

File Name: Supplementary Data 3
Description: Methylation analysis at 17q21.31.

File Name: Supplementary Data 4
Description: Enrichment analysis of 375 differentially methylated genes.

File Name: Supplementary Data 5
Description: Information used for pathway analysis.

File Name: Supplementary Data 6
Description: Post-hoc Power Analysis.
